# Ligand discrimination between active and inactive activation loop conformations of Aurora-A kinase is unmodified by phosphorylation†
†Electronic supplementary information (ESI) available. See DOI: 10.1039/c8sc03669a


**DOI:** 10.1039/c8sc03669a

**Published:** 2019-03-04

**Authors:** James A. H. Gilburt, Paul Girvan, Julian Blagg, Liming Ying, Charlotte A. Dodson

**Affiliations:** a Molecular Medicine , National Heart & Lung Institute , Imperial College London , SAF Building , London SW7 2AZ , UK; b Cancer Research UK Cancer Therapeutics Unit , The Institute of Cancer Research , 15 Cotswold Road , Sutton , Surrey SM2 5NG , UK; c Department of Pharmacy and Pharmacology , University of Bath , Claverton Down , Bath BA2 7AY , UK . Email: c.a.dodson@bath.ac.uk

## Abstract

Structure-based drug design is commonly used to guide the development of potent and specific enzyme inhibitors. Many enzymes – such as protein kinases – adopt multiple conformations, and conformational interconversion is expected to impact on the design of small molecule inhibitors. We measured the dynamic equilibrium between DFG-in-like active and DFG-out-like inactive conformations of the activation loop of unphosphorylated Aurora-A alone, in the presence of the activator TPX2, and in the presence of kinase inhibitors. The unphosphorylated kinase had a shorter residence time of the activation loop in the active conformation and a shift in the position of equilibrium towards the inactive conformation compared with phosphorylated kinase for all conditions measured. Ligand binding was associated with a change in the position of conformational equilibrium which was specific to each ligand and independent of the kinase phosphorylation state. As a consequence of this, the ability of a ligand to discriminate between active and inactive activation loop conformations was also independent of phosphorylation. Importantly, we discovered that the presence of multiple enzyme conformations can lead to a plateau in the overall ligand *K*_d_, despite increasing affinity for the chosen target conformation, and modelled the conformational discrimination necessary for a conformation-promoting ligand.

## Abstract 2

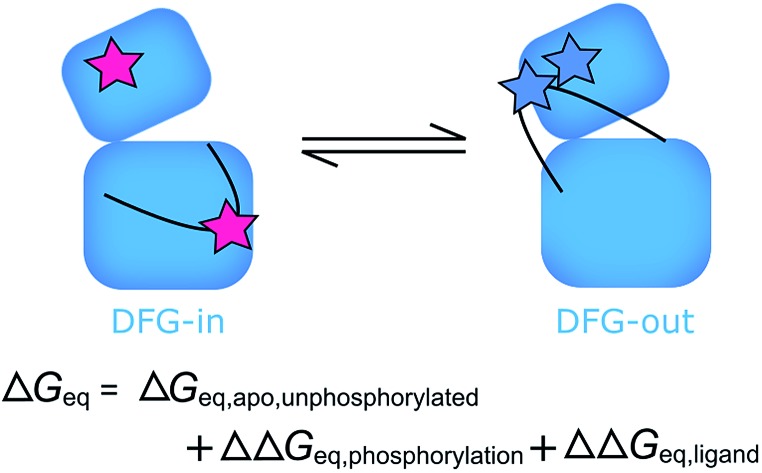
Activation loop phosphorylation changes the position of equilibrium between DFG-in-like and DFG-out-like conformations but not the conformational preference of inhibitors.

## 

Protein kinases are key regulators of the living cell and inhibition of kinase activity is a therapeutic strategy in multiple diseases.^1^–^7^ Many kinases are regulated by phosphorylation of a specific serine, threonine or tyrosine residue on a region of the kinase known as the activation loop and the orientation of this loop is critical to kinase activity.^8^,^9^ In an active kinase, the activation loop is oriented such that the catalytic residues are aligned, a magnesium ion is coordinated in the ATP binding site and the protein substrate binding site is complete. Rotation of the activation loop through 180° disrupts these interactions and results in the kinase adopting an inactive conformation. Most small molecule kinase inhibitors bind in the ATP-binding site, an activation loop-dependent pocket, or both^4^,^10^ and so the conformation of the kinase is believed to be crucial to inhibitor affinity.

Previous work on phosphorylated Aurora-A^11^,^12^ and on p38α^13^ has provided direct evidence that the two conformations of the activation loop are in equilibrium and established that the position of this equilibrium can be modulated by the binding of ligands to these kinases. However, the effect of phosphorylation on the dynamics of the activation loop of any kinase, the position of equilibrium and the interaction between kinase phosphorylation and ligand-binding in determining loop conformation remain largely unknown. These questions are important in medicinal chemistry because static target structures are widely used to drive the development of new kinase inhibitors *via* structure-based drug design. They are also important in translating optimized compounds to biological assays because the phosphorylation state of the physiological kinase, and thus the potential effect of a small molecule inhibitor on kinase function, varies with cellular context. Understanding the dynamic relationship between phosphorylation state, kinase conformation and kinase ligand binding will enable a better mechanistic understanding of the cellular phenotype of kinase inhibitors in different tissues, disease types and at different spatiotemporal points within the cell cycle.

Here we set out to answer these questions using the cancer-associated mitotic kinase Aurora-A as a model. Aurora-A is the target of several drug discovery programs^14^,^15^ and is a well-characterized exemplar for robust biophysical measurement.^11^,^12^,^16^ We discovered that the activation loop of unphosphorylated Aurora-A was also in dynamic equilibrium and that the population of the inactive loop conformation was increased compared with the phosphorylated kinase. This is contrary to the results of a recent FRET study.^17^ We discovered that phosphorylation increased the residence time of the activation loop in the active conformation, leaving that of the inactive conformation unchanged. Compared with phosphorylated kinase, the position of equilibrium in the presence of kinase ligands was shifted towards the inactive conformation for all ligands tested. This shift was associated with a ligand-dependent free energy change which was independent of phosphorylation state, underlining the independence of these two mechanisms of regulation.^16^ We determined that the activation loop of Aurora-A adopts one of only two major conformations and modelled the relationship between the conformation-specific *K*_d_ (commonly used in structure-based drug design) and overall *K*_d_ measured in standard biophysical assays.

## Results

### Unphosphorylated Aurora-A occupies a predominantly inactive activation loop conformation

**Fig. 1:**
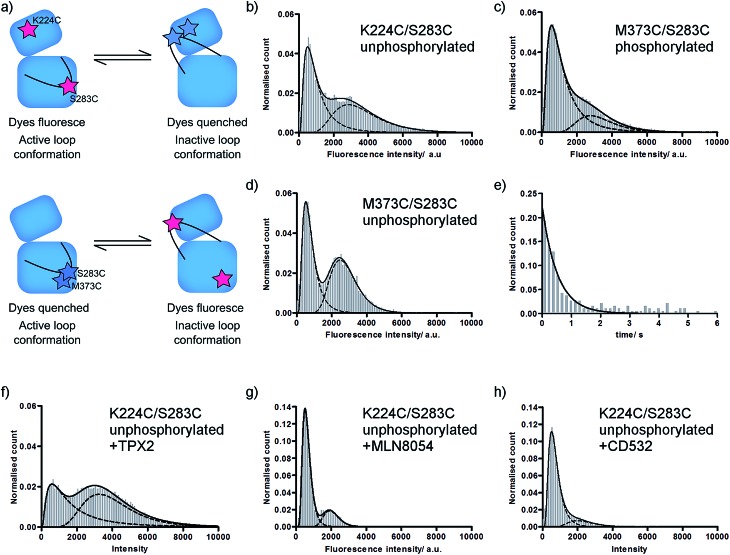
Fluorescence histograms for TMR-labelled Aurora-A. (a) Cartoon of assay used. (b–d) Fluorescence intensity histograms for (b) unphosphorylated K224C/S283C; (c) phosphorylated M373C/S283C; (d) unphosphorylated M373C/S283C; (e) dwell time histogram for unphosphorylated K224C/S283C; (f–h) fluorescence intensity histograms for unphosphorylated K224C/S283C in the presence of (f) 5 μM TPX2; (g) 10 μM MLN8054; (h) 10 μM CD532. All ligand concentrations are expected to be saturating. Note different *y*-axis scales for MLN8054 and CD532. Fitted peak modes and widths, experimental number of molecules, and experimental number of frames included are listed in ESI Table SI.† Example data traces for all conditions shown in ESI Fig. S2.†

We have previously used single molecule fluorescence spectroscopy to monitor the interconversion of the activation loop of phosphorylated Aurora-A kinase between active and inactive conformations.^11^ In our assay, two TMR dye molecules are site-specifically attached to the Aurora-A kinase domain such that they report on the conformation of the loop: one dye is attached to the activation loop, one is situated on the N-lobe of the kinase (αD helix; K224C/S283C). When the loop is in an active conformation the labelled residues are around 40 Å apart and the dyes fluoresce, when the loop is in an inactive conformation the labelled residues are 15 Å apart and fluorescence is quenched (Fig. 1a upper; ESI Fig. S1a†).^11^,^18^

In order to estimate the distance between the two dye molecules themselves we calculated the dye-accessible volume for each protein conformation (ESI Fig. S1b†).^19^ From these calculations we determined the distances between the mean position of the TMR dyes to be 45 Å (active loop conformation) and 18 Å (inactive loop conformation). Our approach offers considerable advantages over traditional FRET methods since our method reports on small distance changes: the transition from quenched to high fluorescence is reported to occur for a residue–residue distance change of 16 Å to 21 Å.^18^ We have calculated the expected FRET values for conformational change in Aurora-A using modelled mean dye positions for the conventional FRET pair Alexa488/Alexa568 (*R*_0_ = 62 Å). These calculations indicate that we would expect to measure high FRET under all circumstances (100% FRET for the inactive activation loop conformation and 87% for the active activation loop conformation).

In order to determine the effect of phosphorylation on the conformational equilibrium of Aurora-A we used our assay to measure a single molecule intensity histogram for unphosphorylated kinase (Fig. 1b). From the ratio of areas of the two fitted peaks, we determined that 52 ± 1% of unphosphorylated kinase occupies an inactive conformation (Table 1). This is greater than the 23% we measured for the phosphorylated kinase^11^ and is consistent with the lower catalytic activity of the unphosphorylated kinase.^16^

**Table 1:** Occupancy of Aurora-A conformations under different conditions

Construct	Phosphorylation state	Ligand	Inactive loop^*a*^/%	Active loop^*a*^/%	*K*_eq_ ^*b*^	Δ*G*_inactive–active_ at 25 °C^*c*^/kcal mol^–1^
K224C/S283C	Phosphorylated	Apo^*d*^	23 ± 1	77 ± 1	0.3 ± 0.1	0.7
		TPX2^*d*^	14 ± 2	86 ± 2	0.2 ± 0.1	1.1
		MLN5084^*d*^	43 ± 2	57 ± 2	0.7 ± 0.1	0.2
		CD532^*d*^	64 ± 1	36 ± 2	1.8 ± 0.1	–0.4
	Unphosphorylated	Apo	52 ± 1	46 ± 2	1.1 ± 0.1	–0.1
		TPX2	46 ± 2	56 ± 4	0.8 ± 0.1	0.1
		MLN8054	77 ± 1	21 ± 1	3.7 ± 0.2	–0.8
		CD532	83 ± 1	13 ± 1	6.3 ± 0.6	–1.1
M373C/S283C	Phosphorylated	Apo	25 ± 2	72 ± 1	0.3 ± 0.1	0.6
	Unphosphorylated	Apo	51 ± 2	47 ± 1	1.1 ± 0.1	–0.0

^*a*^Error reported is propagated fitting error from histograms.

^*b*^ *K*_eq_ = [inactive loop]/[active loop]. Reported error is propagated from the fitting error of the histograms.

^*c*^Δ*G*_inactive–active_ = –*RT* ln(*K*_eq_). The propagated error on Δ*G*_inactive–active_ is ≤0.1 kcal mol^–1^.

^*d*^Data from ref. 11 and included here for ease of comparison.

### Aurora-A activation loop occupies only two major conformations

X-ray structures of Aurora-A show the activation loop in one of only two major conformations (ESI Fig. S1c†). However, it remains possible that the loop occupies one or more additional conformations, perhaps at low occupancy, which have not been captured in protein crystals. Each of our experiments reports on a binary quantity – whether or not the two TMR molecules are close enough together for the dyes to quench – and our original K224C/S283C construct formally reports only on whether the activation loop is or is not in the inactive conformation. This means that we would obtain an identical high fluorescence signal for a single active conformation and for multiple conformations in which the reporter dyes remain far apart in space (*e.g.* an active conformation and a second inactive conformation; or a disordered loop sampling multiple intermediate conformations).

In order to probe the number of conformations adopted by the Aurora-A activation loop we designed a second dye-labelled Aurora-A construct (M373C/S283C; Fig. 1a lower; ESI Fig. S1a†). If the activation loop adopts only two major conformations, we expect the observed fluorescence intensity distribution of M273C/S283C to be the exact inverse of K224C/S283C. Any difference between the results of the two constructs would represent the population of a putative third activation loop conformation. Our measured single molecule histograms for M373C/S283C and K224C/S283C are indeed the inverse of each other for both phosphorylated and unphosphorylated kinase (Fig. 1c, d and Table 1), indicating that the activation loop of Aurora-A adopts only two major conformations: one active and one inactive. Each of these conformational ensembles is likely to contain structural heterogeneity, but the extent of this will be constrained by the quenching radius of the dye pair.^18^

### Phosphorylation increases the residence time in the active conformation

In order to determine the dynamics of interconversion in the unphosphorylated kinase, we measured the dwell-time of the inactive conformation (Fig. 1e). *k*_active_, the fitted rate constant for inactive to active conformations, was 2.1 ± 0.1 s^–1^, within experimental error of the value previously measured for phosphorylated kinase (2.3 ± 0.1 s^–1^).^11^ However *k*_inactive_, the calculated rate constant for adopting the inactive conformation, was 2.4 ± 0.2 s^–1^, more than three times faster than that for phosphorylated kinase (0.7 ± 0.2 s^–1^). In other words, the residence time of the loop in the inactive conformation (0.5 ± 0.1 s) is unaltered by phosphorylation, while the residence time of the loop in the active conformation is increased by a factor of more than three (0.4 ± 0.1 s unphosphorylated; 1.5 ± 0.5 s phosphorylated).

Consistent with our observation that *k*_active_ is unchanged between phosphorylated and unphosphorylated enzyme, X-ray structures of Aurora-A do not show phosphorylation-dependent contacts in the inactive activation loop conformation (*e.g.* PDBs ; 2WTV, ; 4J8M). We expected that the increased residence time of the phosphorylated kinase in the active conformation would be explained by the classical pThr–Arg interactions found in the active conformation of the activation loop of HRD kinases (in Aurora-A, these would be electrostatic interactions between pThr288 and Arg255 (HRD motif), Arg286 (activation loop) and Arg180 (αC helix); ESI Fig. S3a†). However, an alignment of the Aurora-A structures in the PDB indicates that these contacts are only observed in the structures of Aurora-A bound to its protein activator TPX2 (; 3E5A, ; 1OL5, ; 3HA6),^20^–^22^ to N-Myc (; 5G1X)^23^ or to mimics of these (; 5LXM).^24^ We noticed that 24 of the 25 PDB structures with an active conformation activation loop in which pThr288 was modelled were crystallized in the same crystal form (*P*6_1_22; ESI Table SII†). In this form, pThr287 and pThr288 on the activation loop pack within ∼10 Å of Gln127 and a cluster of positively charged residues (Arg179, Arg180, Arg255, Arg286) on two symmetry related molecules, potentially explaining the apparent solvent-exposure of the pThr and the non-classical conformation of the activation loop in these structures (ESI Fig. S1c†). Our results, which are of ligand-free kinase in solution, suggest that under our experimental conditions either pThr288 adopts the classical HRD kinase interactions or that the phosphorylated activation loop is stabilized in an active-like conformation by interactions observed in some of the ATP-bound X-ray structures (pThr288 with Lys143 (glycine-rich loop) in ; 5DNR (space group *P*4_1_2_1_2) or pThr287 with Arg180 and Arg255 in ; 5DT3; ESI Fig. S3b and c†).^25^,^26^

### Changes to the conformational equilibrium upon ligand binding are independent of phosphorylation state

Unphosphorylated Aurora-A can bind both activator protein (TPX2)^16^,^27^ and small molecule inhibitors. We monitored the position of conformational equilibrium for unphosphorylated Aurora-A in the presence of saturating quantities of TPX2, MLN8054 and CD532 using the K224C/S283C reporter construct (Fig. 1f–h and Table 1). For all ligands, the position of equilibrium was shifted towards the inactive conformation compared with that of the same ligand bound to phosphorylated enzyme.^11^

We next computed the ratio of equilibrium constants between apo and ligand-bound kinase for both unphosphorylated and phosphorylated enzyme (ESI Table SII†). To our surprise, this change was independent of phosphorylation state. Since1
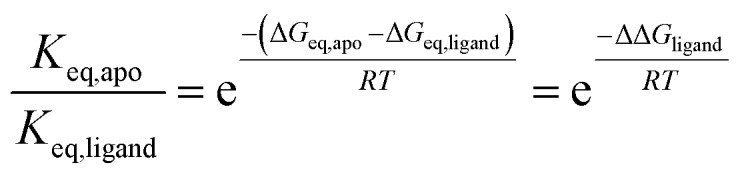
where Δ*G*_eq,apo_ is the free energy difference between active and inactive conformations in the absence of ligand, Δ*G*_eq,ligand_ the free energy difference in the presence of ligand, and ΔΔ*G*_ligand_ = Δ*G*_eq,apo_ – Δ*G*_eq,ligand_; this means that the change in relative stabilities of active and inactive activation loop conformations upon ligand binding (ΔΔ*G*_ligand_) is also independent of kinase phosphorylation. A similar analysis comparing the effect of phosphorylation revealed that the free energy change associated with shifting the position of equilibrium upon phosphorylation (ΔΔ*G*_phosphorylation_) is independent of ligand (ESI Table SII†).

Formally, this means that the free energy difference between active and inactive conformations of Aurora-A can be calculated for any combination of ligand and phosphorylation state as follows:

2 Δ*G*_eq_ = Δ*G*_eq,apo,unphosphorylated_ – ΔΔ*G*_phosphorylation_ – ΔΔ*G*_ligand_

where Δ*G*_eq,apo,unphosphorylated_ is the equilibrium free energy difference between active and inactive activation loop conformations for the unliganded unphosphorylated enzyme and either ΔΔ*G*_phosphorylation_ or ΔΔ*G*_ligand_ may be zero for unphosphorylated or unliganded kinase.

### Ligand discrimination between active and inactive conformations is independent of phosphorylation state

We have shown previously that ligand discrimination, the ability of a ligand to discriminate between active and inactive activation loop conformations of Aurora-A, is related to the conformational equilibrium constants in the presence and absence of ligand:^11^3
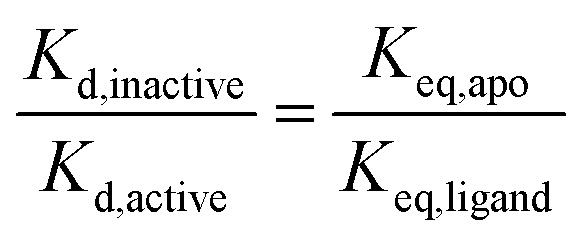
where *K*_d,active_ is the dissociation constant for the ligand from the active conformation of the kinase, *K*_d,inactive_ is the dissociation constant for the ligand from the inactive conformation of the kinase and ligand discrimination is defined as the ratio *K*_d,inactive_/*K*_d,active_. Since our experimental results show that *K*_eq,apo_/*K*_eq,ligand_ is independent of phosphorylation for TPX2, MLN8054 and CD532, the ability of these ligands to discriminate between active and inactive conformations of Aurora-A in their binding must also be independent of activation loop phosphorylation. This is logical because none of the ligand binding sites includes pThr288, and is important because it indicates that development of Aurora-A inhibitors should optimize their binding to a specific conformation of the kinase (active or inactive) rather than a specific phosphorylation state.

### Achieving a single conformation of Aurora-A with a ligand requires a large difference in binding affinity for each conformation

None of our experiments to date has achieved 100% of either active or inactive conformation of Aurora-A and we were curious about the properties of a theoretical ligand which might achieve this. Inducing the inactive activation loop conformation of the Aurora-A activation loop with small molecule ligands has been proposed as a therapeutic strategy in N-Myc driven cancers since this conformation is expected to release Aurora-A-bound N-Myc into the cellular pool for degradation.^23^,^28^–^30^

We calculated the ligand discrimination (eqn (3)) necessary to achieve three different values of *K*_eq,ligand_ for different phosphorylation states in the presence and absence of TPX2 (Table 2). The values of *K*_eq,ligand_ were chosen to be equivalent to populations of the active conformation of 1%, 5% and 10%, which we considered *a priori* to be plausible target endpoints for a ligand-driven conformational perturbation. Our calculations show that achieving 1% active conformation for the unphosphorylated enzyme requires a ligand with more than an 80-fold difference in binding affinity for the inactive *versus* active Aurora-A activation loop conformations, rising to several hundred-fold for the enzyme bound to TPX2.

**Table 2:** Calculated ligand discrimination necessary to achieve stated population of inactive conformation

	99% inactive conformation^*a*^		95% inactive conformation^*a*^		90% inactive conformation^*a*^	
	Discrimination^*b*^	Fold preference^*c*^	Discrimination^*b*^	Fold preference^*c*^	Discrimination^*b*^	Fold preference^*c*^
Phosphorylated kinase	0.003	331	0.016	64	0.033	30
Unphosphorylated kinase	0.011	88	0.059	17	0.125	8
Phosphorylated + TPX2	0.002	608	0.009	117	0.018	55
Unphosphorylated + TPX2	0.012	81	0.064	16	0.135	7

^*a*^99% inactive conformation equivalent to *K*_eq_ = 99; 95% inactive conformation equivalent to *K*_eq_ = 19; 90% inactive conformation equivalent to *K*_eq_ = 9.

^*b*^Ligand discrimination (eqn (3)) required to achieve stated percentage of inactive conformation.

^*c*^Fold preference of ligand for inactive conformation required to achieve stated percentage of inactive conformation. Fold preference = 1/ligand discrimination.

### Phosphorylation and TPX2 change the relative stabilities of Aurora-A ground and transition states

**Fig. 2:**
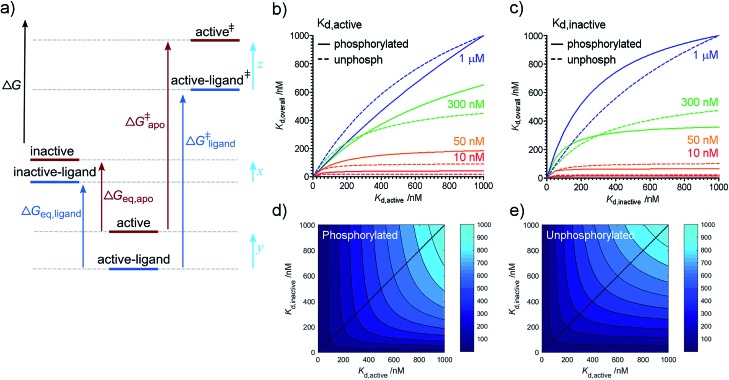
Free energy diagram and partitioning *K*_d_. (a) Free energy diagram showing relationship between equilibrium and kinetic measurements. Inactive: kinase in inactive activation loop conformation; active: kinase in active activation loop conformation; active^‡^ and active-ligand^‡^: transition state complexes (activated enzyme–substrate complexes). The ligand-bound kinase has been shown arbitrarily as more stable than the apo kinase. A similar diagram can be drawn for phosphorylation. ΔΔ*G*_ligand_ = Δ*G*_eq,apo_ – Δ*G*_eq,ligand_ which can be rearranged to show that ΔΔ*G*_ligand_ = *x* – *y*. ΔΔ*G*‡ligand = Δ*G*‡apo – Δ*G*‡ligand = *z* – *y*. (b–d) Relationship between overall and conformation-specific dissociation constants for Aurora-A. (b) Variation in *K*_d,overall_ with *K*_d,active_ at fixed values of *K*_d,inactive_ (values of *K*_d,inactive_ indicated by colored text and lines). (c) Variation in *K*_d,overall_ with *K*_d,inactive_ at fixed values of *K*_d,active_ (values of *K*_d,active_ indicated by colored text and lines). Solid lines – phosphorylated kinase; dashed lines – unphosphorylated kinase. (d) Contour plot showing *K*_d,overall_ (values indicated by shade of blue) as a function *K*_d,active_ and *K*_d,inactive_ for phosphorylated Aurora-A. (e) Contour plot showing *K*_d,overall_ (values indicated by shade of blue) as a function of *K*_d,active_ and *K*_d,inactive_ for unphosphorylated Aurora-A. Blue dotted line in panels (d) and (e) indicate the diagonal *K*_d,inactive_ = *K*_d,active_ and is included to guide the eye.

Our previous kinetic measurements discovered that each of Aurora-A phosphorylation and TPX2 binding makes an energetically independent contribution to catalysis.^16^ While both equilibrium and kinetic measurements show energetic independence between phosphorylation and TPX2 binding, it is important to recognize that ΔΔ*G*_ligand_ and ΔΔ*G*_phosphorylation_ reflect changes in the energetics of ground state conformations while the kinetic measurements reflect changes in the energetics of transition state species relative to ground state (Fig. 2a).

Conformational interconversion is fast compared with the catalytic rates we have previously measured^16^ and we initially wondered whether quantities such as ΔΔ*G*_ligand_ and ΔΔ*G*_phosphorylation_ would explain the observed differences in the rate of catalysis. Comparison with our previous measurements shows that this is not the case, indicating that TPX2 and phosphorylation contribute to changes in the relative stabilities of transition states (*z*–*y* in Fig. 2a) in addition to changes in ground state (*x*–*y* in Fig. 2a). Note that it is only possible to determine *relative* changes in species stability (*z*–*y* or *x*–*y*), not *absolute* changes (quantities *x*, *y*, or *z*). Physical mechanisms by which this is achieved are likely to include changes in solvation^12^ and structural heterogeneity within the two major conformational ensembles resolved in our experiments (*e.g.* small changes in the position of active site residues not detected by our assay which could easily contribute to changes in the *K*_d_ and the kinetic parameters *K*_m_ and *k*_cat_).

### Structure-based ligand optimization may stall due to ligand affinity for an alternative conformation

Our results, here and previously,^11^ show that both phosphorylated and unphosphorylated Aurora-A adopt the same activation loop conformations, albeit in different proportions. Since the physiological cell contains pools of phosphorylated and of unphosphorylated protein, we wondered which phosphorylation state was preferable to use in a drug discovery program for biophysical screens of *K*_d_, and how the dissociation constant reported in a screening assay would vary with the phosphorylation state used. We also wondered how optimizing the dissociation constant against a single structural conformation of the protein would influence the overall *K*_d_ measured.

We can rearrange eqn (S15) and (S18) in ref. 11 to show that4
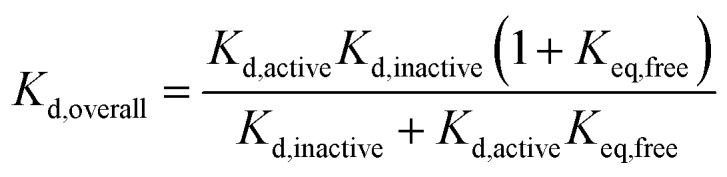
and we used our measured values of *K*_eq,free_ to calculate *K*_d,overall_ for each phosphorylation state of Aurora-A at different values of *K*_d,active_ and *K*_d,inactive_ (Fig. 2b–d). As expected, compounds which bind more tightly to the active activation loop conformation (*i.e. K*_d,active_ < *K*_d,inactive_) bind more tightly to the phosphorylated kinase (Fig. 2b and c). This means that differential binding of a compound between phosphorylated and unphosphorylated Aurora-A is expected to highlight preferential binding for one conformation of the activation loop.

We can use eqn (4) to create a surface where the height of the surface is the overall dissociation constant, *K*_d,overall_, plotted as a function of conformation-specific dissociation constants (Fig. 2d and e). During lead optimization, a medicinal chemist aims to reduce the overall dissociation constant of the hit compound by making changes to its binding properties. These changes may affect the affinity of the ligand for either conformation of the kinase – essentially medicinal chemistry design moves the hit compound across the surface, always aiming to move downhill towards the minimum (ESI Fig. S4†). The distance moved across the surface is an indicator of the likelihood that a small chemical change will bring about this effect (small changes in conformation-specific dissociation constant – represented by short distances in defined directions – are more likely to be achieved).

Having derived this surface analytically, we decided to use it to test the extent to which it can provide a rationale for some commonly adopted medicinal chemistry practices. In a structure-based drug design project, a hit compound will usually be optimized against one of the two conformations of the target. Our model supports this decision since from nearly all points on the surface, the shortest distance to a high affinity compound is parallel or nearly parallel to the *x* or *y* axis (ESI Fig. S5†). It is common practice to optimize binding to the conformation of the target to which the compound binds preferentially (if known). Again, our model broadly supports this decision. Interestingly, our analysis reveals that the line on the surface along which a point is equidistant (in terms of *K*_d,active_ and *K*_d,inactive_) from a specified contour line varies with the value of the contour line (ESI text and eqn (S8)†). However as the overall affinity increases, this equidistant line approaches the diagonal *K*_d,inactive_ = *K*_d,active_ leading to broad support with common chemical practice (ESI Fig. S6†).

Our model also enables us to make predictions. There are several plateau regions in Fig. 2b–d, where changes in the conformation-specific binding affinity bring about very little change in the overall dissociation constant (*e.g.* in Fig. 2b, a five-fold change in *K*_d,active_ from 1 μM to 200 nM along the orange line (*K*_d,inactive_ = 50 nM) brings little change in *K*_d,overall_). The way in which these plateau regions arise depends on the value of *K*_eq_ (ESI Fig. S7†). Should lead optimization using a single target structure stall in a drug discovery program, this may be because the compounds tested lie in one of these affinity regions. In this case, we predict that optimizing against the second conformation will improve overall binding affinity (in the previous example, moving from orange to red lines and making a five-fold change in *K*_d,inactive_ from 50 nM to 10 nM). Such a switch in medicinal chemistry design strategy would benefit from structural knowledge of the second conformation and provides further support for wider structural biology investigation and computational chemistry modelling of alternative protein binding site conformations.^31^–^33^

## Conclusions

We have previously shown that phosphorylated Aurora-A is in dynamic equilibrium between active and inactive activation loop conformations and that the position of this equilibrium can be modified by the binding of the protein activator TPX2 and of small molecule inhibitors.^11^ Our experiments here show that unphosphorylated Aurora-A is also in dynamic equilibrium between active and inactive activation loop conformations, and that both phosphorylated and unphosphorylated enzyme interconvert between only two major conformations.

Ligand-binding and phosphorylation both change the position of the Aurora-A conformational equilibrium. Each is associated with a specific free energy change which is independent of the other leading us to discover that ligand discrimination between active and inactive activation loop conformations is independent of kinase phosphorylation. We have not yet measured 100% of the inactive activation loop conformation under any conditions and we predict the properties of a small molecule which would bring this about (more than an 80-fold difference in binding affinity between active and inactive Aurora-A activation loop conformations in the absence of other factors).

Since phosphorylated and unphosphorylated Aurora-A sample the same conformational ensembles, our results indicate that small molecule Aurora-A inhibitors will target both phosphorylation states, even if the initial optimization was carried out against one. Carrying out structure-based drug design against a single static enzyme conformation may lead to an apparent plateau in the experimental dissociation constant. In this case, optimizing against the second kinase conformation could increase overall compound potency. Of particular importance is the notion that considering ligand affinity for both conformational states of a kinase may be useful in rational medicinal chemistry design. This concept also provides additional impetus for both experimental and computational investigation of inactive and active protein-ligand complex conformations.

## Methods

Aurora-A point mutants were generated by Quikchange (Agilent), and His-tagged Aurora-A and TPX2 (residues 1–43) expressed and purified as previously described.^11^,^34^ Unphosphorylated Aurora-A was obtained by co-expression with untagged λ-phosphatase.^34^,^35^

Protein labelling, single molecule spectroscopy and data fitting were carried out as previously described.^11^ Briefly, His-tagged K224C/S283C/C290A/C393A or S283C/C290A/M373C/C393A Aurora-A kinase domain (residues 122–403) was reacted with excess 5′-tetramethylrhodamine iodoacetamide (TMRIA) at 4 °C overnight. The reaction was quenched with DTT and unreacted dye separated from protein by desalting. Labelling efficiency (ESI Table SIV†) was calculated as a percentage of available labelling sites (*i.e.* 200% is full occupancy of all sites) based on absorption at 280 nm and 514 nm and assumes random labelling. Protein was frozen at –80 °C until use.

MLN8054 was purchased from Selleck Chemicals and CD532 synthesized according to the literature.^30^ All inhibitors were equilibrated with sample (in imaging buffer) for at least 10 min before data collection.

Protein samples were tethered to the PEGylated surface of a home-built sample cell *via* their His-tag (long linker sequence for free movement of protein), a biotinylated anti-His antibody, neutravidin, and biotinylated PEG (present at a low percentage in PEGylation step). The sample cell was washed with imaging buffer (0.3 mg mL^–1^ BSA, 50 mM Tris–HCl pH 7.5, 200 mM NaCl, 5 mM MgCl_2_, 10% glycerol, 5 mM protocatechuic acid, 0.1 μM protocatechuate 3,4-dioxygenase, 1% DMSO and 5 mM Trolox) before data acquisition to remove non-specifically bound protein.

Single molecule TIRF measurements were made at room temperature using a ∼2.0 mW 514 nm laser in a home-built optical setup. Fluorescence was captured by a CoolView EM 1000 camera using an 80 ms per frame capture speed and 2 × 2 pixel binning with 500 frames per video. Data was processed using custom written IDL (background subtraction, spot identification) and Matlab (trace selection, frame binning) scripts. Intensity histograms were fit to the sum of two log normal distributions and the dwell time histogram fit to a single exponential decay using Prism (www.graphpad.com).

Transitions between high and low intensity states occur much faster than our frame rate (within a single frame). Transitions are clearly distinct from noise (noise is much smaller than the intensity change due to transition) and boundaries for dwell time analysis were determined by visual inspection. The percentage of all molecules transitioning within the acquisition time is given in ESI Table SV.†

Inter-dye distances were determined using FPS software^19^ (freely available from ; www.mpc.uni-duesseldorf.de) to calculate the dye-accessible volume and mean dye positions for TMR-labelled Aurora-A. Dyes were modelled as ellipsoids with radii of 7.1 Å, 4.3 Å, 1.8 Å (TMR), 5.0 Å, 4.5 Å, 1.5 Å (Alexa 488) and 8.1 Å, 4.2 Å, 2.1 Å (Alexa568) with each dye being attached to the amino acid C_α_ by a flexible linker of length 8.3 Å and width 4.5 Å. The accessible volume of each dye was modelled using PDBs 2DWB (active conformation) and ; 2WTV (inactive conformation) in which the residues for dye attachment had been manually mutated to glycine. This mutation enables changes in the orientation of the C_β_–S bond (which would otherwise be prohibited by selecting a single rotamer in a Cys mutation) and also prevents an artefactual reduction in the dye-accessible volume caused by presence of the original Lys or Ser side-chain. Raw and partially processed data are deposited in Zenodo (DOI: 10.5281/zenodo.2555379).

## Conflicts of interest

J. B. is a former employee of The Institute of Cancer Research, which has a commercial interest in the development of Aurora-A inhibitors. P. G. was funded by an Imperial College-AstraZeneca Innovation Fund award to C. A. D.. J. A. H. G. and L. Y. have no competing financial interests.

## Supplementary Material

Supplementary information
