# Supplementary material for: Ligand discrimination between active and inactive activation loop conformations of Aurora-A kinase is unmodified by phosphorylation
Source: Chem Sci. 2019 Mar 4;10(14):4069–76. doi: 10.1039/c8sc03669a (PMC6461105; doi:10.1039/c8sc03669a)
Supplement: Supplementary file 1 [file SC-010-C8SC03669A-s001.pdf]

This journal is © The Royal Society of Chemistry 2019

Supplementary text

Calculating the threshold between optimizing for active and inactive enzyme conformations

On a surface characterized by equation (4) (here rewritten as equation (S1) in terms of x, y and z for ease of notation), a point (x,y) is on the surface a distance of Δx from the contour line z = C (C = constant) in a direction parallel to the x-axis, and a distance of Δy from the contour line z = C in a direction parallel to the y-axis.

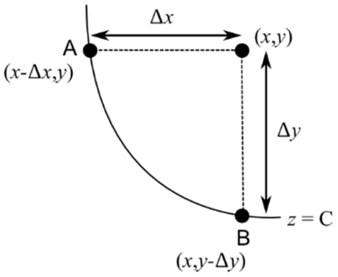

Thus coordinates of point A are (x - Δx, y) and point B are (x,y - Δy).

$$z = \frac{x \cdot y(1+K_{eq})}{x \cdot K_{eq} + y} \tag{S1}$$

Substituting coordinates into (S1), at A

$$C = \frac{(x-\Delta x)\,y\left(1+K_{eq}\right)}{(x-\Delta x)K_{eq} + y} \tag{S2}$$

Rearranging,

$$\Delta x = \frac{y\left[x\left(1+K_{eq}\right)-C\right]-x \cdot C \cdot K_{eq}}{y\left(1+K_{eq}\right)-C \cdot K_{eq}} \tag{S3}$$

Similarly, at B

$$C = \frac{x(y-\Delta y)\left(1+K_{eq}\right)}{x \cdot K_{eq} + y - \Delta y} \tag{S4}$$

and thus

$$\Delta y = \frac{y\left[x\left(1+K_{eq}\right)-C\right]-x \cdot C \cdot K_{eq}}{x\left(1+K_{eq}\right)-C} \tag{S5}$$

For the set of points where Δx=Δy, (S3) and (S5) can be equated and rearranged as below

$$\left[y\left[x\left(1+K_{eq}\right)-C\right]-x \cdot C \cdot K_{eq}\right]\left[(y-x)\left(1+K_{eq}\right)+C\left(1-K_{eq}\right)\right]=0 \tag{S6}$$

The two solutions to this equation are

$$y = \frac{x \cdot C \cdot K_{eq}}{x\left(1+K_{eq}\right)-C} \tag{S7}$$

and

$$y = x - \frac{C\left(1-K_{eq}\right)}{1+K_{eq}} \tag{S8}$$

where (S7) is the equation of the contour line z = C, and (S8) is a straight line with slope = 1 which intersects z = C at $y = x\sqrt{K_{eq}}$.

Estimating the inclusion of two co-localized single-labelled molecules in our analysis (the false positive rate)

All single molecule traces included in our analysis exhibited two-step (and only two-step) photobleaching to background-subtracted zero, with each photobleaching step reducing the original intensity by around half. This selection step ensures that all analyzed traces originate from molecules possessing two fluorophores (*ie* double-labelled protein samples), and excludes most single-labelled proteins (originating from incomplete labelling). Nevertheless, two single-labelled protein molecules co-localized within the same diffraction-limited spot also fulfil this criteria. We refer to the mistaken inclusion of two co-localized single-labelled molecules in our analysis as our false positive rate.

It is important to note that false positive traces will never transition to a quenched intensity (will never show dynamic behavior) since TMR quenching requires the two dyes to be very close to (within ~16 Å of) each other [18]. Thus false positive traces will only be included in our fluorescence intensity histograms, not our dwell time histograms (our kinetic measurements). For the same reason, false positive traces will only contribute to the population of the unquenched peak in the intensity histogram.

In order to estimate our false positive rate we reanalyzed the data from our single-labelled control experiments [11]. The experimental conditions for these data are identical to those reported in this paper, except that the samples used have only one labelling site (K224C or S283C depending on the data set). Since all molecules in these experiments are single- (or un-) labelled, any traces showing two photobleaching steps must be due to two co-localized molecules

As expected, there were several traces for our single-labelled protein samples which had two photobleaching steps, and which otherwise met all of our standard criteria for inclusion as an experimental trace. For K224C this was 3 ± 1 % of total traces, for S283C this was 2 ± 1 % of total traces (values reported are mean and standard deviation of five experiments). By eye, these two experiments had different immobilization densities (K224C an unusually dense field of view, S283C a less dense 'normal' field of view), giving us confidence that the co-localization rate we measured is a good estimate of the co-localization rate in our other experiments. For these two experiments, around 10 % of all traces met the inclusion criteria (including the presence of a single photobleaching step) for the histograms which we previously published [11] meaning that the ratio of co-localized:dispersed molecules for K224C (dense field of view) is 3:10 and for S283C (normal density) is 1:5.

In our experiments with double-labelled protein, only co-localization events involving two single-labelled protein molecules contribute to our false positive rate. Co-localization of two double-labelled proteins (4 photobleaching steps) or one single and one double-labelled protein (3 photobleaching steps) would already be excluded from analysis. We therefore used our labelling efficiencies to calculate the number of single-labelled molecules as a proportion of the total number of labelled proteins and scaled the co-localized part of the co-localized:dispersed ratio accordingly. We assumed that the ratio of co-localized:dispersed molecules for slides of similar immobilization density remained constant throughout our experiments, and therefore used this rescaled ratio to estimate our false positive rate (Supplementary table SIV).

However, there is no simple relationship between our false positive rate and the error introduced by this on the reported values from our intensity histograms. In other words, a 5% change in the number of traces included in a peak will not result in a 5% change in the area of that peak. Additionally, each experimental trace is of a different length (since photobleaching is stochastic), so a 5% change in the number of traces does not translate into a 5% change in the number of frames included.

In order to estimate the effect of our false positive rate on our experimental results, we compared our results for K224C/S283C and M373C/S283 constructs. Both phosphorylated and unphosphorylated experiments report the same conformational distributions, yet these samples have enormously different labelling efficiencies (Supplementary table SIV). If our false positive rate had a large influence on our histogram percentages, we would expect phosphorylated and unphosphorylated experiments to report different degrees of agreement between K224C/S283C and M373C/S283C constructs as there are considerable differences across our samples in the probability of a co-localisation event being from two single-labelled molecules (0.33 – 0.86; Supplementary table SIV). Since experimental histograms for K224C/S283C and M373C/S283C give conformational distributions within experimental error of each other for both phosphorylated and unphosphorylated kinase, we are encouraged that the impact of our false positive rate is low.

Supplementary table SI: Fitted parameters from single molecule intensity histograms [a]

		Low fluorescence peak		High fluorescence peak		High fluorescence mode / low fluorescence mode	Number of molecules analysed	Total number of frames analysed
	ligand	Mode	Width parameter	Mode	Width parameter			
M373C/S283C phosphorylated	Apo	565 ± 83	0.75 ± 0.02	2774 ± 106	0.35 ± 0.03	4.9 ± 0.3	204	26297
M373C/S283C unphosphorylated	Apo	520 ± 7	0.56 ± 0.01	2480 ± 25	0.29 ± 0.01	4.8 ± 0.3	79	9312
K224C/S283C unphosphorylated	Apo	499 ± 9	0.73 ± 0.03	2858 ± 78	0.41 ± 0.02	5.7 ± 0.4	167	24212
	TPX2	591 ± 19	0.94 ± 0.05	3252 ± 43	0.39 ± 0.01	5.5 ± 0.4	175	13532
	MLN8054	506 ± 3	0.41 ± 0.01	1906 ± 32	0.22 ± 0.02	3.8 ± 0.2	52	7398
	CD532	531 ± 4	0.50 ± 0.01	2103 ± 109	0.28 ± 0.05	4.0 ± 0.3	54	5858

[a] Errors are fitting errors (width parameter) or values propagated from fitting error (mode and ratio of modes)

Supplementary table SII: Comparing equilibria for activation loop phosphorylation and for different ligands

Ligand	Ratio of K_{eq} [a]		$\Delta\Delta G_{\text{inactive-active}}$ [b]		Contribution of phosphorylation	
	Phosphorylated	Unphosphorylated	Phosphorylated	Unphosphorylated	Ratio of K_{eq} [c]	$\Delta\Delta G_{inactive\text{-}active}$ /kcal mol^{-1} [d]
Apo	-	-	-	-	0.3 ± 0.1	0.8 ± 0.1
TPX2	1.7 ± 0.2	1.4 ± 0.1	-0.3 ± 0.1	-0.2 ± 0.1	0.2 ± 0.1	0.9 ± 0.1
MLN8054	0.4 ± 0.1	0.3 ± 0.1	0.6 ± 0.1	0.7 ± 0.1	0.2 ± 0.1	1.0 ± 0.1
CD532	0.2 ± 0.1	0.2 ± 0.1	1.1 ± 0.1	1.0 ± 0.1	0.3 ± 0.2	0.7 ± 0.1

[a] Ratio of $K_{eq} = K_{eq,apo} / K_{eq,ligand}$

[b] $\Delta\Delta G_{inactive\text{-}inactive} = \Delta G_{inactive\text{-}active,apo} - \Delta G_{inactive\text{-}active,ligand}$

[c] Ratio of $K_{eq} = K_{eq,phosphorylated} / K_{eq,unphosphorylated}$

[d] $\Delta\Delta G_{active\text{-}inactive} = \Delta G_{inactive\text{-}active,phosphorylated} - \Delta G_{inactive\text{-}active,unphosphorylated}$

Supplementary table SIII: Crystal forms of Aurora-A PDB structures in which residue 288 is explicitly modelled as phosphothreonine

PDB ID	Space group	Ligand
1ol5	$P2_12_12_1$	TPX2
3e5a	$C222_1$	TPX2
3ha6	$P3_121$	TPX2
5g1x	$P3_221$	N-Myc
5lxm	$P3_121$	TPX2 proteomimic
1ol7	$P6_122$	
2w1c	$P6_122$	
4bn1	$P6_122$	
4ceg	$P6_122$	
5orl	$P6_122$	
5orn	$P6_122$	
5oro	$P6_122$	
5orp	$P6_122$	
5orr	$P6_122$	
5ors	$P6_122$	
5ort	$P6_122$	
5orw	$P6_122$	
5orx	$P6_122$	
5ory	$P6_122$	
5orz	$P6_122$	
5os0	$P6_122$	
5os1	$P6_122$	
5os3	$P6_122$	
5os4	$P6_122$	
5os5	$P6_122$	
5os6	$P6_122$	
5osd	$P6_122$	
5ose	$P6_122$	
5osf	$P6_122$	
5dnr	$P4_12_12$	

Supplementary table SIV: Estimating false positive rate

	K224C/S283C phosphorylated	K224C/S283C unphosphorylated	M373C/S283C phosphorylated	M373C/S283C unphosphorylated
Labelling efficiency (% of sites)	120 %	31 %	58 %	80 %
Double-labelled molecules (% of all molecules in sample [a])	36 %	2 %	8 %	16 %
Single-labelled molecules (% of all molecules in sample [a])	48 %	26 %	41 %	48 %
Single-labelled molecules (% of all labelled molecules)	57 %	93 %	84 %	75 %
Probability of a co-localization event being from two single-labelled molecules	0.33	0.86	0.71	0.56
Estimated false positive rate [b]	6 %	15 %	12 %	10 %

[a] Assuming random labelling of sites

[b] Assuming that the ratio of co-localized : dispersed molecules among traces meeting the criteria for analysis remains similar to that measured in S283C control (*ie* 1:5). False positive rate = probability of trace being due to co-localization (1/6) * probability of co-localization event resulting from two single-labelled molecules

Supplementary table SV: Percentage of molecules interconverting within acquisition time [a]

	Ligand	Percentage of molecules interconverting [b]
M373C/S283C phosphorylated	Apo	32
M373C/S283C unphosphorylated	Apo	22
K224C/S283C unphosphorylated	Apo	28
	TPX2	20
	MLN8054	9
	CD532	20

[a] Statistics for intensity histograms (Figure 1b-d, f-h).

[b] A molecule was scored as interconverting if a transition from high intensity to quenched intensity (or vice versa) was observed before the first photobleaching event occurred. The reported numbers should be considered a lower bound, since many traces photobleached relatively quickly.

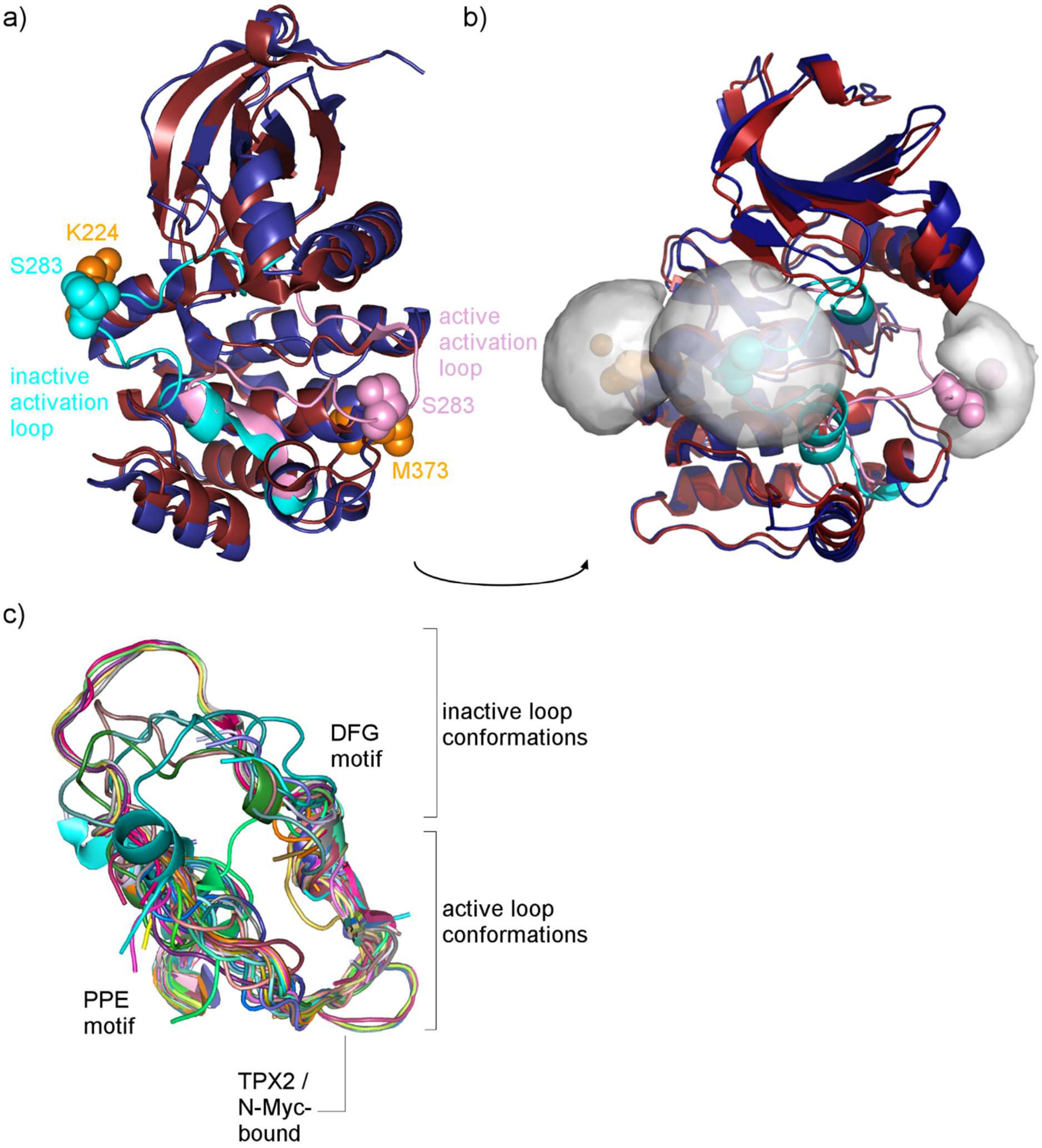

Supplementary figure S1: Two major conformations of the Aurora-A activation loop. a) Aurora-A kinase showing active and inactive conformations of the activation loop and dye labelling sites. Deep red / pink – active kinase (1OL7), activation loop in pink. Deep blue / cyan – inactive kinase (2WTV), activation loop in cyan. Orange – dye labelling positions on kinase surface. Dye labelling sites shown as spheres. b) Aurora-A kinase showing dye-accessible volumes (white surfaces) and mean dye positions (colored sphere within each volume). Backbone atoms of Lys224 and Ser283 also shown as spheres. Distances between mean dye position and C_{α} are 5.5 Å (position 224), 4.4 Å (position 283, inactive activation loop) and 7.3 Å (active activation loop). All colors as panel (a). Kinase rotated slightly from orientation in panel (a) for clarity in inactive activation loop conformation. c) Aligned

activation loops of the 134 human Aurora-A structures in PDB (loop is not modelled in all structures). Residues shown are 274-299 (DFG-PPE motifs). The activation loop of the kinase bound to TPX2 or N-Myc shows a slightly different conformation at the tip of the loop. One activation loop appears to adopt a conformation which is neither active nor inactive. In this structure (PDB 5EW9) the phosphate of pThr287 is around 4 Å from the N1 of His187 in a symmetry related molecule, suggesting that stabilization of this conformation is a crystallographic artefact.

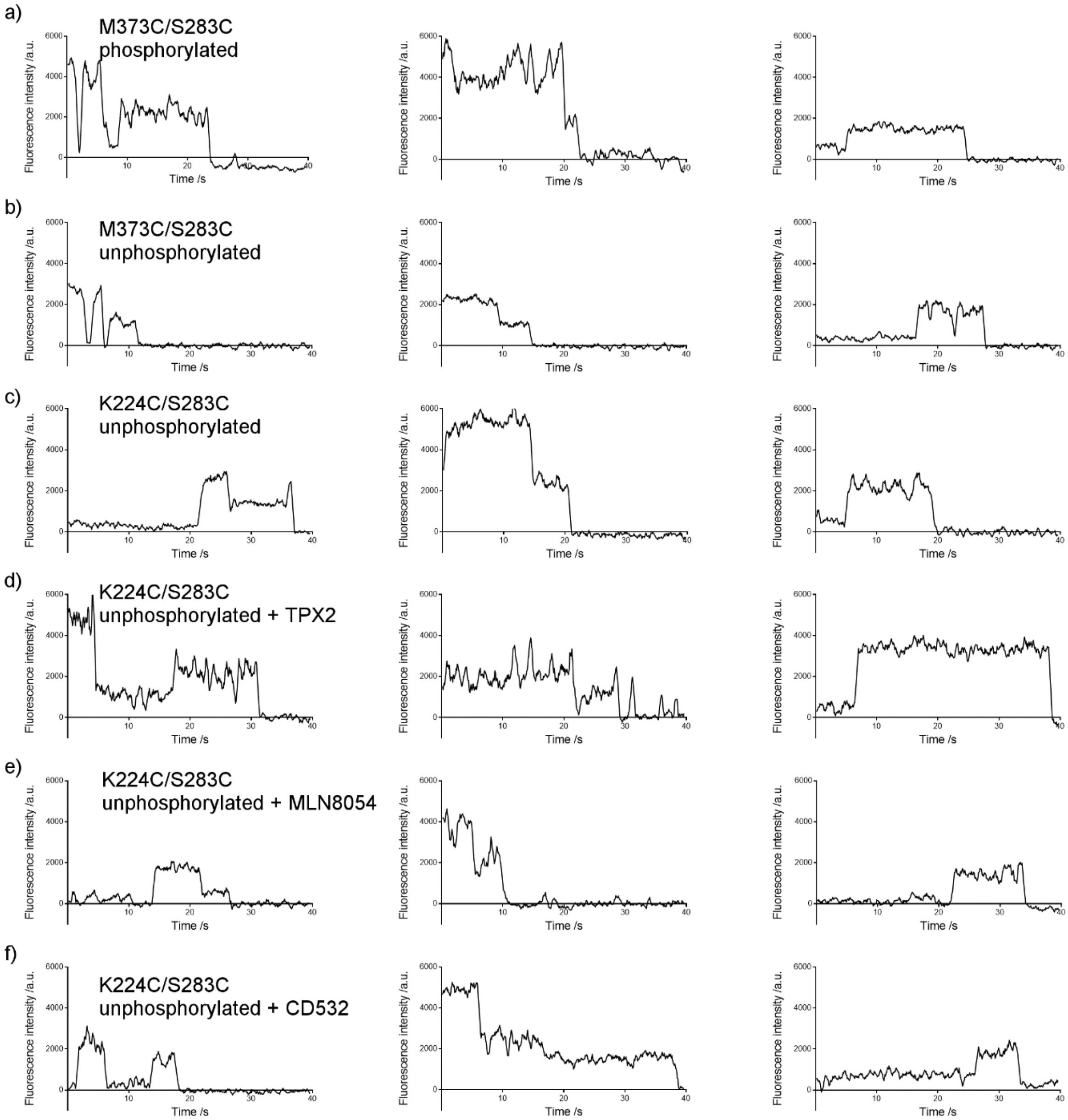

Supplementary figure S2: Example data for each condition. *Left:* Molecule shown transitions between quenched and unquenched (or vice versa). *Middle:* Molecule shown does not transition and is initially unquenched (high fluorescence). *Right:* Molecule shown does not transition and initially quenched (low fluorescence). In these panels, fluorescence increases after first photobleaching event since fluorescence of remaining dye is no longer quenched. All traces have been smoothed with a three-point moving average.

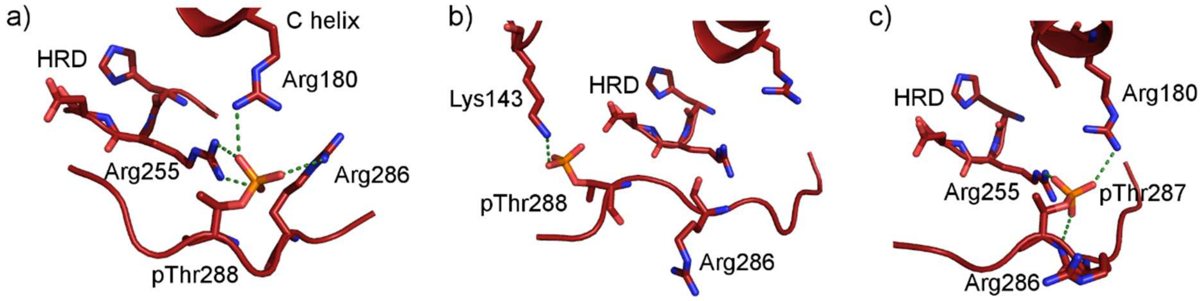

Supplementary figure S3: Phosphorylation-dependent hydrogen bonds stabilizing the active conformation of Aurora-A. a) Classical HRD kinase pThr hydrogen bonding pattern observed in TPX2, N-Myc and TPX2 mimic-bound structures only (1OL5 shown here); b) non-canonical hydrogen bond between glycine-rich loop (Lys143) and pThr288 observed in ATP-bound structures 5DNR and 5DN3; c) pThr277 can form many of the canonical hydrogen bonds usually made by pThr288 (5DT3 and 4BN1).

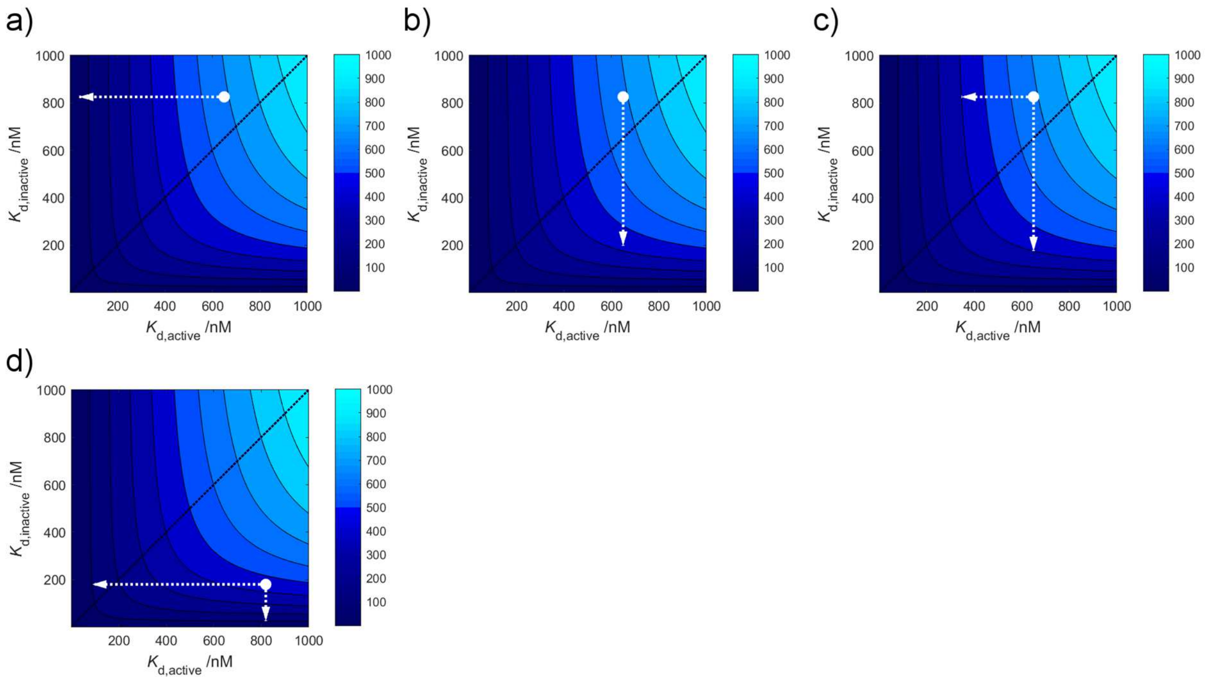

Supplementary figure S4: Walking a compound over a binding surface. a) A compound with initial $K_{d,overall}$ of 680 nM ($K_{d,active}$ = 650 nM, $K_{d,inactive}$ = 825 nM) is improved for binding to the active activation loop conformation only (final $K_{d,overall}$ = 50 nM; $K_{d,active}$ = 39 nM; $K_{d,inactive}$ = 825 nM). b) The same compound (initial $K_{d,overall}$ = 680 nM, $K_{d,active}$ = 650 nM, $K_{d,inactive}$ = 825 nM) is improved for binding to the inactive activation loop conformation only (final $K_{d,overall}$ = 425 nM, $K_{d,active}$ = 650 nM, $K_{d,inactive}$ = 197 nM). c) To achieve the same $K_{d,overall}$ (here 400 nM), the change needed in $K_{d,active}$ (625 nM to 346 nM; white horizontal line) is much less than the change needed in $K_{d,inactive}$ (825 nM to 175 nM; white vertical line). d) Starting at a different point on the surface ($K_{d,active}$ = 820 nM, $K_{d,inactive}$ = 180 nM, $K_{d,overall}$ = 450 nM) and aiming for a $K_{d,overall}$ of 100 nM, optimizing for binding to the active conformation would require a much bigger change in $K_{d,active}$ (from 820 nM to 88 nM; white horizontal line) compared with optimizing for binding to the inactive activation loop conformation ($K_{d,inactive}$ from 180 nM to 25 nM; white vertical line). All panels: Colors of contour map indicate values of $K_{d,overall}$ and contour lines are shown every 100 nM. Contour map plotted for phosphorylated kinase (K_{eq} = 0.3).

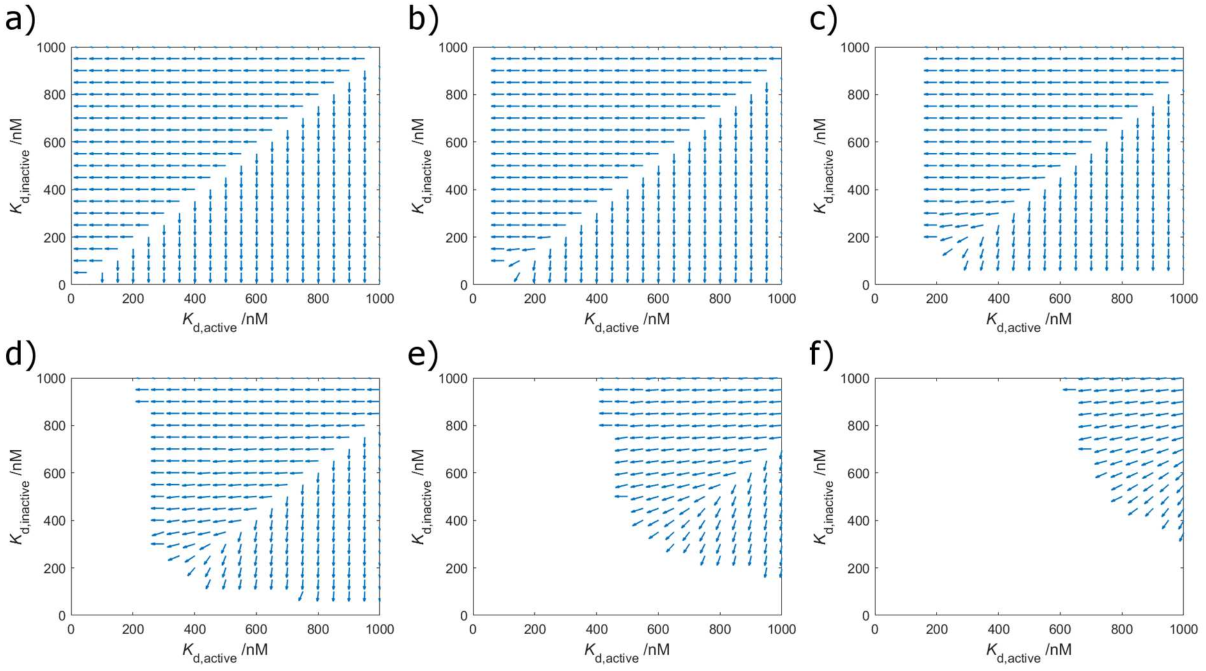

Supplementary figure S5: Optimizing ligand affinity for a single protein conformational state (active or inactive) is the best strategy to achieve a potent ligand overall (panel a). In the hypothetical situation of designing weaker ligands, it may be better to optimize binding to both protein conformations simultaneously, particularly for initial hit compounds which have similar initial affinities for each kinase conformation (diagonal arrows in panels b-f). Plots show the direction of the shortest vector distance between any point on the surface and stated contour line. The direction of the arrow indicates whether the shortest distance to the target $K_{d,overall}$ is via optimising $K_{d,active}$ (horizontal arrows), $K_{d,inactive}$ (vertical arrows), or a combination of the two. Arrows only plotted for points where the initial $K_{d,overall}$ is greater than the contour line reached. a) $K_{d,overall}$ = 1 nM. b) $K_{d,overall}$ = 100 nM. c) $K_{d,overall}$ = 200 nM. d) $K_{d,overall}$ = 300 nM. e) $K_{d,overall}$ = 500 nM. f) $K_{d,overall}$ = 700 nM. Arrows plotted every 50 nM and normalised to be the same length. K_{eq} = 0.3.

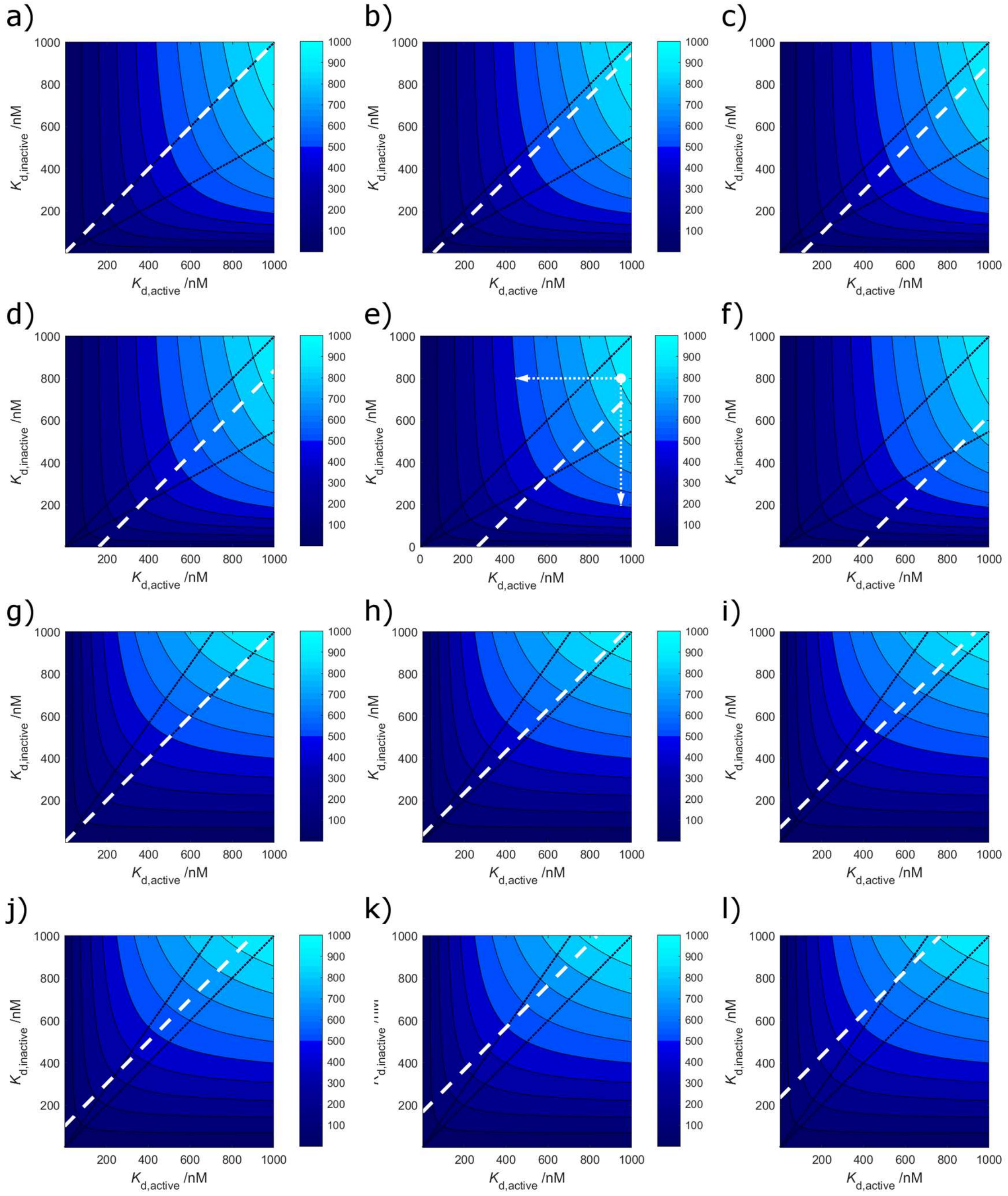

Supplementary figure S6: When designing a potent ligand, the ligand should be optimized to bind the conformation of the kinase to which it has greatest affinity (panels a&g). In the hypothetical situation of designing a less potent ligand, it may be more productive to optimize binding to the conformation of the kinase to which it has initially *least* affinity. This depends on the position of the starting ligand on the surface relative to dashed white line (shown in figure). For ligands which are located on the surface between the dashed white line and the diagonal $K_{d,inactive} = K_{d,active}$, the shortest distance to the target affinity (target contour line) is not by optimizing binding to the conformation to which the initial ligand binds tightest. *Eg* in panel (e), the initial compound binds more tightly to the inactive conformation ($K_{d,active}$ = 950 nM, $K_{d,inactive}$ = 800 nM) but the distance to the contour line $K_{d,overall}$ = 500 nM is shorter along the x-axis (optimizing $K_{d,active}$) than along the y-axis (optimizing $K_{d,inactive}$). Interestingly, the position of the dashed white line (the line along which optimization against active or inactive conformations is equidistant from the stated contour line) changes with the overall potency being targeted. a-f) Contour plots drawn for phosphorylated kinase

(K_{eq} = 0.3). a) $K_{d,overall}$ = 1 nM. b) $K_{d,overall}$ = 100 nM. c) $K_{d,overall}$ = 200 nM. d) $K_{d,overall}$ = 300 nM. e) $K_{d,overall}$ = 500 nM. f) $K_{d,overall}$ = 700 nM. g-l) Contour plots drawn for a hypothetical kinase preferentially adopting the inactive activation loop conformation (K_{eq} = 2). g) $K_{d,overall}$ = 1 nM. h) $K_{d,overall}$ = 100 nM. i) $K_{d,overall}$ = 200 nM. j) $K_{d,overall}$ = 300 nM. k) $K_{d,overall}$ = 500 nM. l) $K_{d,overall}$ = 700 nM. The equidistant line (given by equation (S8)) is shown as a white dashed line. The diagonal $K_{d,inactive}$ = $K_{d,active}$ and the line $K_{d,inactive} = K_{d,active}\sqrt{K_{eq,apo}}$ are shown as blue dashed lines to aid the eye. Colors of contour map indicate values of $K_{d,overall}$ and contour lines are shown every 100 nM.

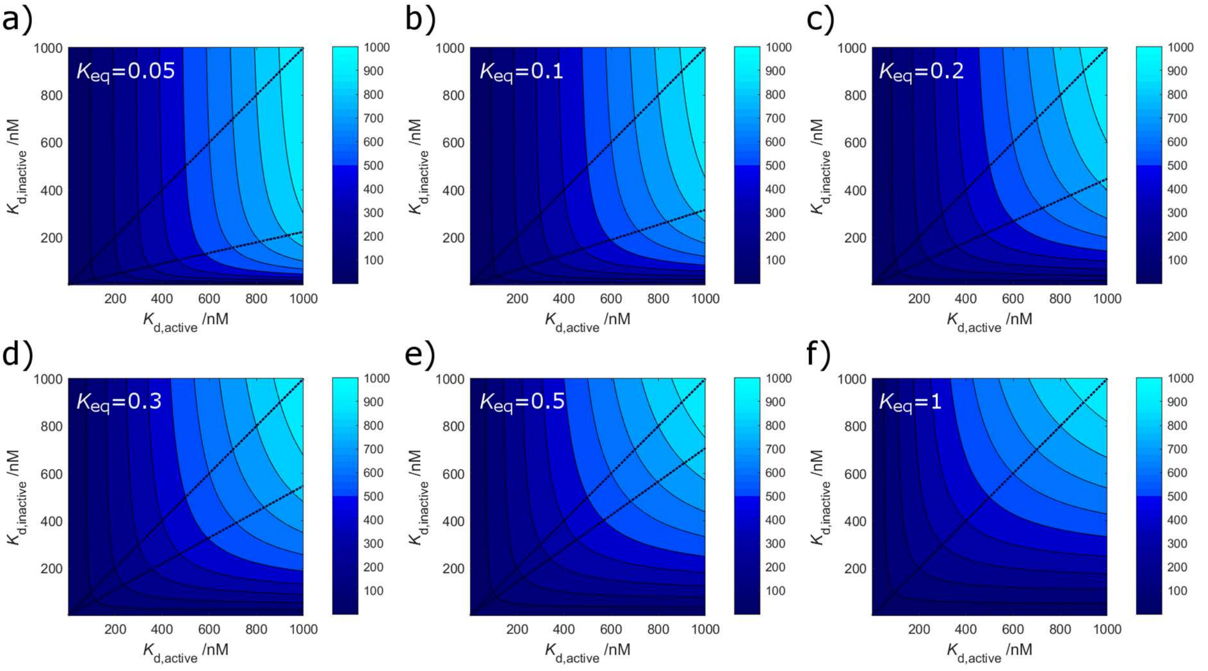

Supplementary figure S7: The position of equilibrium of the kinase determines the appearance of plateau regions (such as those in Figure 2b) because it determines the symmetry of the affinity surface. The more a protein predominantly occupies the active conformation (K_{eq} << 1, *eg* panel a), the more asymmetric the contour lines on the affinity surface. When K_{eq} = 1 (panel f), the surface is completely symmetric. a) K_{eq} = 0.05. b) K_{eq} = 0.1. c) K_{eq} = 0.2. d) K_{eq} = 0.3. e) K_{eq} = 0.5. f) K_{eq} = 1. The diagonal $K_{d,inactive} = K_{d,active}$ and the line $K_{d,inactive} = K_{d,active}\sqrt{K_{eq,apo}}$ are shown as blue dashed lines to aid the eye. Colors of contour map indicate values of $K_{d,overall}$ and contour lines are shown every 100 nM.
